# Vagal Nerve Paraganglioma in a Middle-Aged Woman: A Case Report

**DOI:** 10.7759/cureus.107105

**Published:** 2026-04-15

**Authors:** Hugo E Mora Moreno, Edgar Escorcia Aguirre, María G Maciel García, Bryan G Vasquez Marta, Ulises S Sánchez Guevara

**Affiliations:** 1 Surgery, Hospital General Dr. Miguel Silva, Morelia, MEX; 2 Vascular Surgery, Hospital General Dr. Miguel Silva, Morelia, MEX

**Keywords:** case report, head and neck neoplasms, neuroendocrine tumor, paraganglioma, vagus nerve

## Abstract

Paragangliomas are rare neuroendocrine tumors arising from extra-adrenal paraganglionic tissue, with head and neck involvement representing an uncommon subset. Among these, vagal paragangliomas are particularly rare and typically present as slow-growing, non-functional cervical masses, posing diagnostic and therapeutic challenges due to their proximity to major neurovascular structures. We report the case of a 45-year-old woman with a two-year history of a progressively enlarging right-sided cervical mass without systemic or catecholamine-related symptoms. Imaging studies, including contrast-enhanced computed tomography and computed tomography angiography, revealed a hypervascular tumor within the carotid space with partial encasement of the carotid arteries. The patient underwent complete surgical resection, and histopathological examination confirmed the diagnosis of paraganglioma. Although biochemical evaluation for catecholamine secretion was not performed preoperatively, the patient had an uneventful postoperative course, with preserved vocal function and no evidence of recurrence at one-year follow-up. This case highlights the importance of considering vagal paraganglioma in the differential diagnosis of cervical masses, as well as the critical role of imaging and biochemical assessment in preoperative evaluation. Surgical management remains the mainstay of treatment but carries a significant risk of cranial nerve injury, underscoring the need for careful planning and long-term follow-up.

## Introduction

Paragangliomas are rare neuroendocrine tumors that arise from extra-adrenal paraganglia of the autonomic nervous system and are closely related to pheochromocytomas in terms of origin and biological behavior [1]. These tumors have an estimated annual incidence ranging from 0.04 to 0.95 cases per 100,000 individuals, with increasing detection rates attributed to advances in imaging and biochemical screening [1].

Head and neck paragangliomas represent a small subset of these neoplasms, accounting for less than 0.5% of all head and neck tumors and approximately 3% of all paragangliomas [2]. Among these, vagal paragangliomas are particularly uncommon and typically arise along the course of the vagus nerve within the carotid space. Clinically, they often present as slow-growing, painless cervical masses and are usually non-functional, lacking catecholamine secretion in the majority of cases [3].

Although most head and neck paragangliomas are benign, up to 6-19% may exhibit malignant behavior, defined by the presence of metastasis rather than histological features alone [2]. Additionally, a significant proportion of cases are associated with germline mutations, particularly involving the succinate dehydrogenase (SDH) gene complex, which has important implications for prognosis and familial screening [3].

The diagnostic approach relies on multiple imaging modalities, including contrast-enhanced computed tomography (CT), computed tomography angiography (CTA), magnetic resonance imaging (MRI), and digital subtraction angiography (DSA), which are useful for the evaluation of carotid body and paraganglioma tumors. Among these, DSA has been historically considered the reference standard due to its ability to provide detailed vascular mapping, with reported diagnostic accuracy approaching 100%; however, its use has decreased in favor of less invasive cross-sectional imaging techniques such as CTA and MRI, which are now commonly used for diagnosis and preoperative planning [4,5]. Biochemical evaluation with plasma-free metanephrines is essential to exclude functional tumors prior to surgical intervention, as undiagnosed catecholamine secretion may lead to severe perioperative complications [4].

We present the case of a middle-aged woman with a vagal paraganglioma presenting as a cervical mass, highlighting the clinical, radiological, and surgical features of this rare entity.

## Case presentation

A 45-year-old woman presented with a two-year history of a progressively enlarging right-sided cervical mass, occasionally associated with localized pain. She denied associated symptoms such as fever, night sweats, weight loss, dysphagia, hoarseness, or other constitutional complaints. Her medical history was notable only for chronic use of nonsteroidal anti-inflammatory drugs, with no relevant personal or family history.

The physical examination revealed a well-defined, firm, immobile, and painless mass measuring approximately 8 cm in the right cervical region, with no additional abnormalities. Contrast-enhanced CT of the neck demonstrated a right-sided cervical mass measuring 5 × 3 × 3 cm adjacent to the carotid body. The patient was subsequently referred to the vascular surgery service for further evaluation. Baseline laboratory findings were within normal limits (Table 1).

**Table 1:** Baseline laboratory findings

Parameter	Result	Reference Range
Hemoglobin	14.5 g/dL	12–16 g/dL
Leukocytes	6.0 × 10⁹/L	4.0–10.0 × 10⁹/L
Platelets	368,000/µL	150,000–400,000/µL
Glucose	85 mg/dL	70–100 mg/dL
Prothrombin Time (PT)	16.1 sec	11–14 sec
Activated Partial Thromboplastin Time (aPTT)	25.9 sec	25–35 sec
International Normalized Ratio (INR)	1.21	0.8–1.2

**Figure 1:**
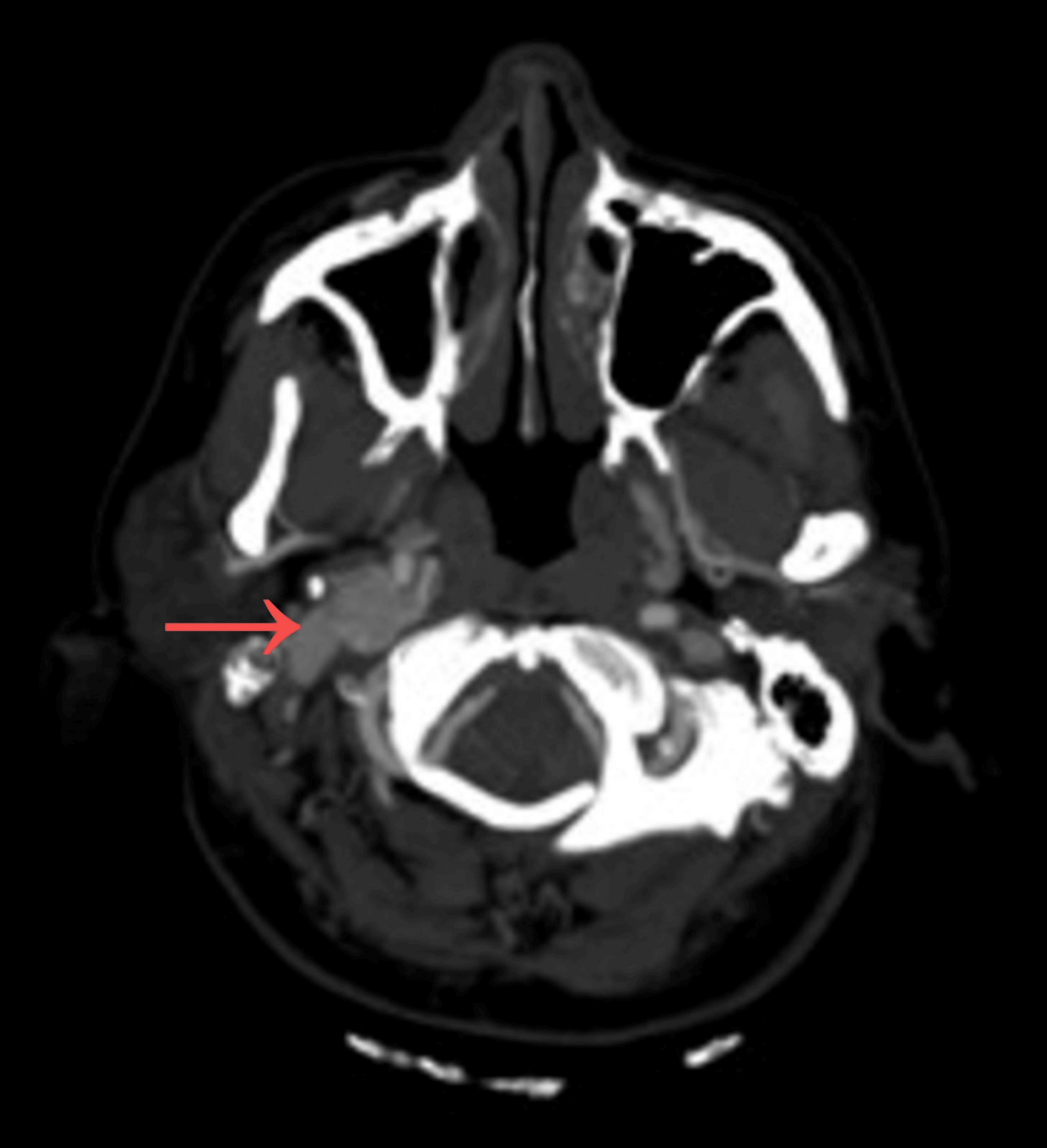
Axial contrast-enhanced computed tomography (CT) scan of the neck demonstrating a right-sided hypervascular mass in the carotid space (arrow), with displacement of adjacent soft tissue structures.

**Figure 2:**
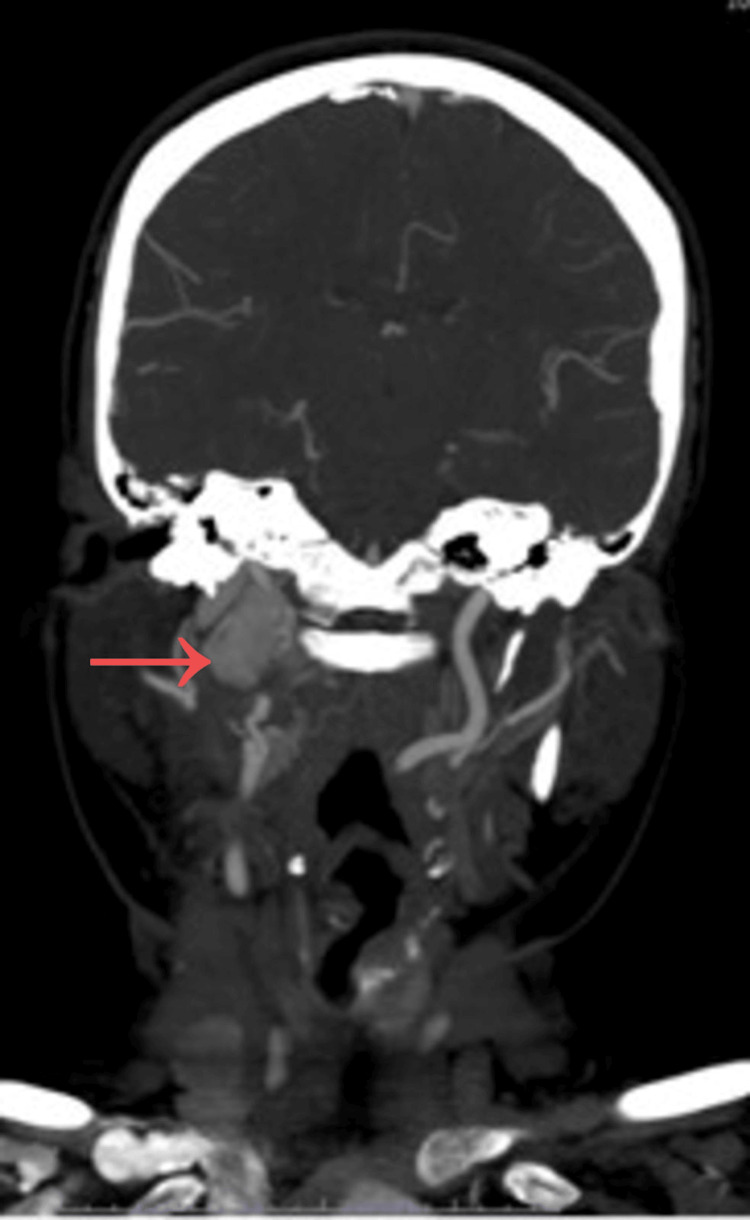
Coronal contrast-enhanced CT scan showing a well-defined mass in the right carotid space (arrow), extending along the vascular axis and causing lateral displacement of surrounding anatomical structures.

Contrast-enhanced CT of the neck revealed a right-sided cervical mass measuring approximately 5 × 3 × 3 cm, located in close proximity to the carotid bifurcation (Figure 1). Coronal imaging further demonstrated the extent of the lesion within the carotid space and its relationship with adjacent anatomical structures (Figure 2).

**Figure 3:**
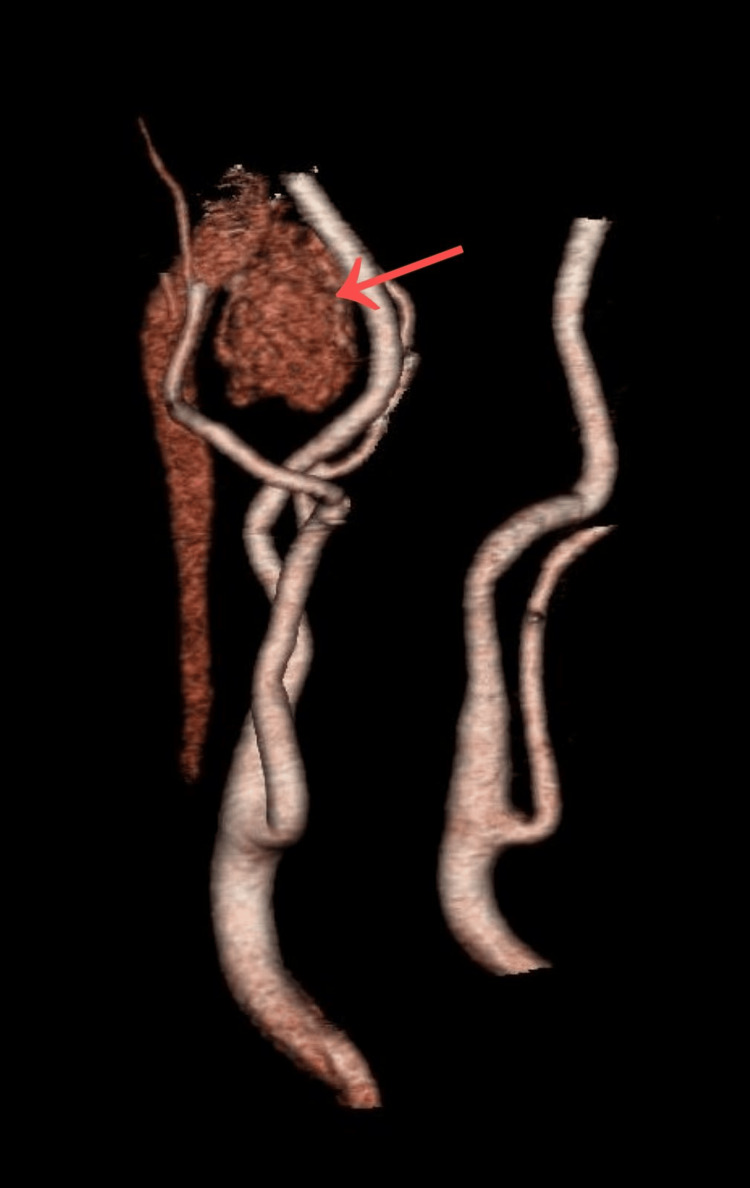
Three-dimensional CT angiography reconstruction illustrating a hypervascular tumor (arrow) with partial encasement of the internal and external carotid arteries, consistent with a paraganglioma.

Subsequent CTA demonstrated a heterogeneous, hypervascular tumor within the right carotid space, causing displacement of adjacent structures. The lesion partially encased both the internal and external carotid arteries and displaced the internal jugular vein (Figure 3), measuring 7.4 × 5.2 × 2.8 cm.

**Figure 4:**
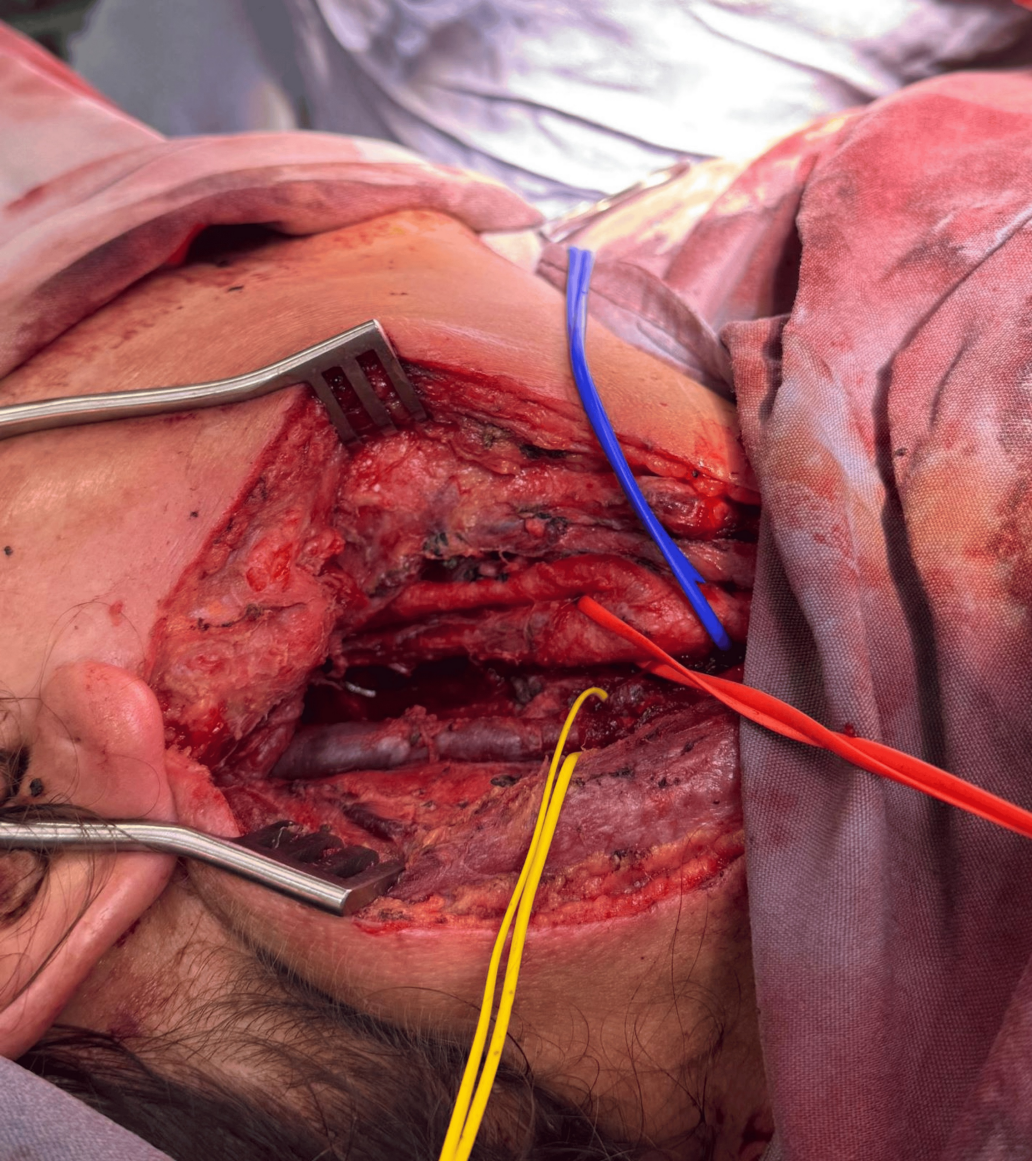
Intraoperative image after tumor resection, showing surgical exposure of the right carotid space. The tumor was in close proximity to major vascular structures, including the carotid arteries (red vascular loop) and adjacent neurovascular elements (yellow vascular loop).

The patient underwent complete surgical excision of the mass. Intraoperative findings confirmed a highly vascularized tumor in close proximity to major vascular and neural structures within the carotid space (Figure 4).

**Figure 5:**
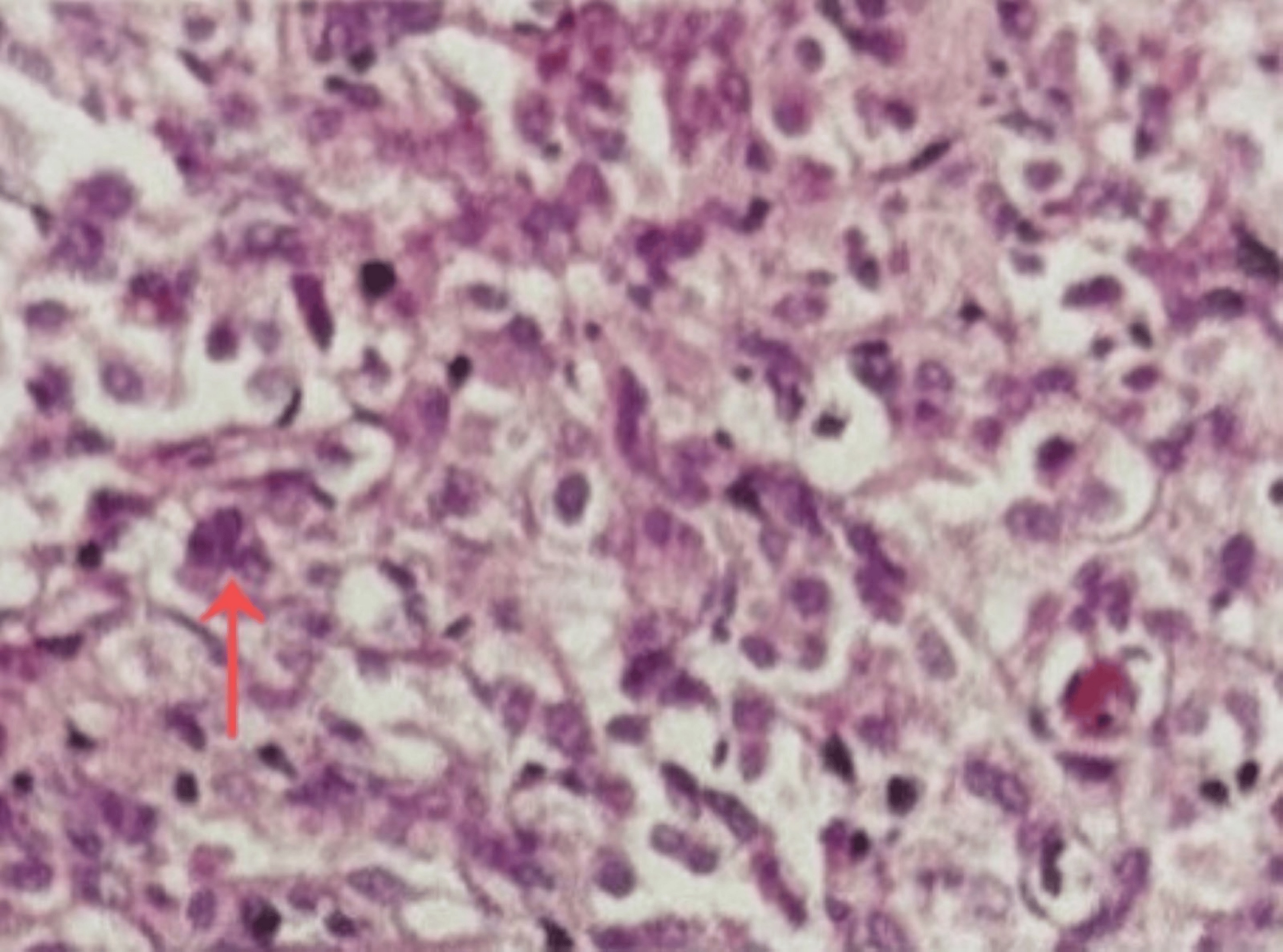
Histopathological examination (hematoxylin and eosin stain) demonstrating nests of epithelioid cells arranged in a characteristic Zellballen pattern (arrow), consistent with paraganglioma (scale bar not available).

Histopathological examination (hematoxylin and eosin stain) demonstrating nests of epithelioid cells arranged in a characteristic Zellballen pattern (arrow), consistent with paraganglioma (Figure 5).

**Figure 6:**
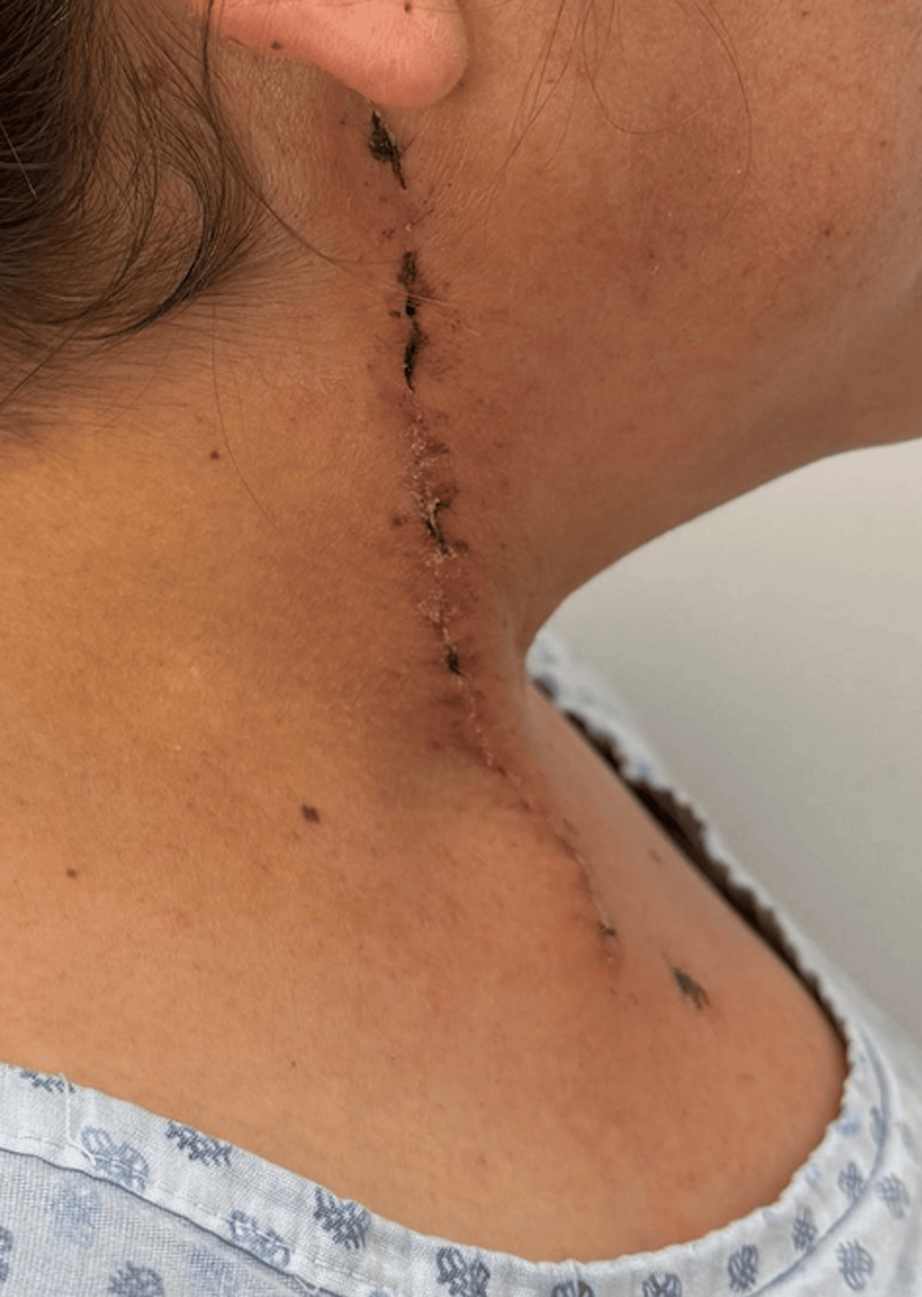
Postoperative clinical image showing the surgical incision site in the right cervical region, with appropriate healing and no evident complications.

At one-year follow-up, the patient remains asymptomatic, with preserved vocal function and no evidence of recurrence. Clinical evaluation revealed no voice changes or symptoms suggestive of vocal cord dysfunction. The surgical site demonstrated appropriate healing without complications (Figure 6).

## Discussion

Vagal paragangliomas are rare neuroendocrine tumors that originate in the paraganglion tissue associated with the vagus nerve, usually located in the carotid space. These tumors are more frequently observed in middle-aged female patients and typically demonstrate slow-growing, indolent behavior consistent with the clinical spectrum of head and neck paragangliomas described in recent literature [6,7]. Their indolent clinical course frequently leads to delayed diagnosis, particularly in non-functional tumors lacking catecholamine-related symptoms.

Imaging studies play a central role in the diagnosis and preoperative assessment of paragangliomas. Contrast-enhanced CT and CTA typically demonstrate a well-defined, hypervascular mass with intense contrast enhancement and characteristic displacement or encasement of adjacent vascular structures [8]. In this case, imaging findings were consistent with these features, demonstrating a hypervascular tumor with partial encasement of the carotid arteries and displacement of the internal jugular vein. These characteristics are critical for surgical planning and risk stratification [9].

Biochemical evaluation is an essential component in the workup of paragangliomas. The measurement of plasma-free metanephrines is considered the most sensitive test for detecting catecholamine-secreting tumors and is recommended prior to surgical intervention [10]. Although most head and neck paragangliomas are non-functional, failure to identify a secreting tumor may result in severe intraoperative hypertensive crises and cardiovascular complications [11]. In the present case, biochemical testing was not performed preoperatively, which represents a deviation from current recommendations and underscores the importance of comprehensive preoperative assessment.

Failure to identify a catecholamine-secreting tumor preoperatively exposes the patient to the risk of a potentially life-threatening intraoperative hypertensive crisis during tumor manipulation, which may result in severe cardiovascular complications such as stroke, myocardial infarction, or death.

Surgical resection remains the mainstay of treatment for vagal paragangliomas; however, it is associated with significant morbidity due to the close anatomical relationship with cranial nerves and major vascular structures [12]. Preservation of the vagus nerve is rarely achieved, with reported rates ranging from 5% to 8%, and postoperative vocal cord paralysis is a common complication [13]. Clinically, this may present as dysphonia, aspiration, and impaired airway protection, significantly affecting quality of life. Notably, our patient did not develop postoperative vocal dysfunction, which represents a favorable outcome given the high risk associated with this procedure.

Alternative and adjunctive treatment options have been increasingly described as alternative or adjunctive treatment modalities in recent literature. Endovascular embolization may be used preoperatively to reduce tumor vascularity and intraoperative blood loss and, in selected high-risk cases, may contribute to tumor control as a primary or palliative strategy [14]. Stereotactic radiotherapy, including fractionated radiotherapy and stereotactic radiosurgery, represents another effective option for unresectable or high-risk tumors, achieving high rates of local control with acceptable morbidity [15].

From a pathological perspective, paragangliomas typically exhibit the characteristic “Zellballen” pattern, consisting of nests of chief cells surrounded by sustentacular cells, confirming their neuroendocrine origin [16]. Although most tumors are benign, malignancy is defined by the presence of metastases rather than histological features, making long-term follow-up essential [17].

Genetic factors also play a crucial role in the pathogenesis of paragangliomas. Mutations in genes encoding subunits of the SDH complex are frequently implicated and are associated with increased risk of multifocal disease and malignancy [18]. Although genetic testing was not performed in this case, its consideration is recommended, particularly in younger patients or those with multifocal or familial disease [19].

This case has several limitations. First, it represents a single case report, which limits the generalizability of the findings. Second, genetic testing for SDH mutations was not performed, which may have provided additional prognostic information. Third, a biochemical evaluation of catecholamine secretion was not conducted prior to surgery. Finally, the one-year follow-up period may be insufficient to detect late recurrence or metastatic disease.

Despite these limitations, this case highlights the importance of a comprehensive diagnostic approach, including imaging and biochemical evaluation, as well as careful surgical planning in the management of vagal paragangliomas.

## Conclusions

Vagal paragangliomas are rare neuroendocrine tumors that should be considered in the differential diagnosis of lateral cervical masses. This case highlights the importance of a comprehensive diagnostic approach, including detailed imaging and appropriate biochemical evaluation to exclude functional tumors prior to surgical intervention. Contrast-enhanced CT and angiographic studies remain valuable diagnostic tools, particularly for defining tumor vascular anatomy and surgical planning, and may help differentiate vagal paragangliomas from carotid body tumors based on lesion location relative to the carotid bifurcation and vascular displacement patterns. Surgical resection remains the cornerstone of treatment but carries a significant risk of cranial nerve injury, particularly involving the vagus nerve. Favorable outcomes, as observed in this patient, depend on careful preoperative planning and meticulous surgical technique. Long-term follow-up is essential given the potential for recurrence and the limitations of histopathological assessment in predicting malignant behavior.

## References

[1] Al Subhi AR, Boyle V, Elston MS (2022). Systematic review: incidence of pheochromocytoma and paraganglioma over 70 years. J Endocr Soc.

[2] Sandow L, Thawani R, Kim MS, Heinrich MC (2023). Paraganglioma of the head and neck: a review. Endocr Pract.

[3] Finkelstein MJ, Stemberger A, McGreal N (2025). Renal paraganglioma: a rare case and review of genetic associations. J Urol Surg.

[4] Blinova NV, Ilovayskaya IA, Chikhladze NM (2024). Diagnosis and management of patients with pheochromocytoma/paraganglioma: consensus of experts of the Russian Medical Society for arterial hypertension and the multidisciplinary group for the diagnosis and treatment of neuroendocrine tumors. Ter Arkh.

[5] Liu J, Mu H, Zhang W (2021). Diagnosis and treatment of carotid body tumors. Am J Transl Res.

[6] Nölting S, Bechmann N, Taieb D (2022). Personalized management of pheochromocytoma and paraganglioma. Endocr Rev.

[7] Crona J, Taïeb D, Pacak K (2017). New perspectives on pheochromocytoma and paraganglioma: toward a precision medicine approach. Nat Rev Endocrinol.

[8] Arya S, Rao V, Juvekar S, Dcruz AK (2008). Carotid body tumors: objective criteria to predict surgical morbidity. Head Neck.

[9] Lee KY, Oh YW, Noh HJ (2006). Extraadrenal paragangliomas of the body: imaging features. AJR Am J Roentgenol.

[10] Lenders JW, Duh QY, Eisenhofer G (2014). Pheochromocytoma and paraganglioma: an endocrine society clinical practice guideline. J Clin Endocrinol Metab.

[11] Fishbein L (2016). Pheochromocytoma and paraganglioma: genetics, diagnosis, and treatment. Hematol Oncol Clin North Am.

[12] Netterville JL, Reilly KM, Robertson D (1995). Carotid body tumors: a review of 30 patients. Laryngoscope.

[13] Makeieff M, Raingeard I, Alric P (2008). Surgical management of carotid body tumors. Ann Surg Oncol.

[14] Helal A, Vakharia K, Brinjikji W (2022). Preoperative embolization of jugular paraganglioma tumors using particles is safe and effective. Interv Neuroradiol.

[15] Fatima N, Pollom E, Soltys S, Chang SD, Meola A (2021). Stereotactic radiosurgery for head and neck paragangliomas: a systematic review and meta-analysis. Neurosurg Rev.

[16] Tischler AS (2008). Pheochromocytoma and paraganglioma: histopathology. Endocr Pathol.

[17] Lloyd RV, Osamura RY, Klöppel G, Rosai J (2017). WHO Classification of Tumours of Endocrine Organs. 4th Edition. IARC.

[18] Dahia PLM (2014). Pheochromocytoma and paraganglioma pathogenesis. Endocr Relat Cancer.

[19] Neumann HP, Young WF Jr, Eng C (2019). Pheochromocytoma and paraganglioma. N Engl J Med.

